# Inventory and composting of yard waste in Serdang, Selangor, Malaysia

**DOI:** 10.1016/j.heliyon.2020.e04486

**Published:** 2020-07-17

**Authors:** Mohammad Hariz Abdul Rahman, Tosiah Sadi, Aimi Athirah Ahmad, Intan Nadhirah Masri, Masnira Mohammad Yusoff, Hasliana Kamaruddin, Nur Alyani Shakri, Mohamad Abhar Akmal Hamid, Rashidah Ab. Malek

**Affiliations:** a Agrobiodiversity & Environment Research Centre, MARDI, 43400 Serdang, Selangor, Malaysia; b Soil & Fertilizer Research Centre, MARDI, 43400 Serdang, Selangor, Malaysia; c Socio Economic, Market Intelligence & Agribusiness Research Center, MARDI, 43400 Serdang, Selangor, Malaysia; d Horticulture Research Centre, MARDI, 43400 Serdang, Selangor, Malaysia

**Keywords:** Yard waste management, Aerated turned composting, Compost quality, Waste planning, Agricultural science, Environmental science, Waste treatment, Environmental management, Waste

## Abstract

Composting of yard waste is one of the waste management approaches in the Malaysian Agricultural Research and Development Institute (MARDI) in Serdang, Selangor, Malaysia. The yard waste inventory was developed in the headquarters’ area and a pilot-scale study was performed on the potential compost product. The total amount of yard waste generated from June 2017 to December 2017 was 16.75 tonnes with an average generation of 0.60 tonnes per week on the fresh weight (f.w.) basis. The collected yard waste consisted of three major characteristics, namely dry leaves, fresh green leaves, and grass cuttings, and a waste estimation technique was applied to determine the composition of these three elements. The acquired information was used to formulate the initial compost mixture. The wastes were then mixed with an appropriate amount of livestock manure and other wastes to obtain the optimum initial C/N ratio, which was then found in the analysis to range between 25:1 and 42:1. Meanwhile, the C/N ratios obtained from the matured compost product were from 10:1 and 15:1. Moreover, most of the compost yield ranged between 50% and 70% (w w^−1^ d.w. basis), while the percentage of the seed germination in the compost was over 95%. The viability of the project was indicated from the economic analysis, with benefit to cost ratio (BCR) values of more than 1. The results also suggested that the large scale composting of yard waste in MARDI was feasible and its applicability is continuous. This technique also fulfilled the objective of producing quality compost, which was suitable for agricultural use.

## 1 Introduction

Yard waste is a type of organic waste produced from the maintenance of gardens and landscaped areas (Shi et al., 2013; Yoshida et al., 2012). Leaves, branches, and grasses are few examples of yard waste that are being produced continuously all year round (Li et al., 2011; Shahudin et al., 2013). Yard waste is high in carbon and ligneous in nature (Hemalatha, 2013), has biodegradable characteristics, and could be decomposed in both aerobic and anaerobic conditions (Boldrin et al., 2011; Yazdani et al., 2012).

The conventional disposal practice in Malaysia involves the landfilling of yard waste (Tarmudi et al., 2012), which is preferable due to low tipping (processing) fees and technological barriers (Samsudina and Dona, 2013). In contrast, conventional landfills are not preferred due to potential environmental problems and slow decomposition process they may cause (Abdel-Shafy and Mansour, 2018). To divert organic waste from landfills, composting is among the implemented strategies (Brown, 2016), which contribute to a high impact at a minimal cost. It also caters for a high amount of waste at any one time.

The Malaysian Agricultural Research and Development Institute (MARDI) is a government research institute based in Serdang, Selangor, Malaysia. The headquarters of MARDI is located in a five hundred hectares of land consisting buildings, landscape, research plots, and reserved land. Although the yard waste generated throughout these areas is managed by transporting the waste to the landfill, MARDI has undertaken measures towards a greener approach since 2017. Such measures included a pilot-scale study looking into the potential of composting the yard wastes and applying them back to the landscape.

In general, composting has several advantages, such as reducing waste volume (Breitenbeck and Schellinger, 2004), stabilising ammonical nitrogen (Li et al., 2013), and destroying the potential of pathogenic threats (Thyagarajan et al., 2013). Specifically, windrow composting has several advantages due to its low capital cost, relatively simple operation, and production of high-quality compost (Vigneswaran et al., 2016). The aerobic composting method, similar to windrow composting, is suitable to be implemented in MARDI. The better aeration rates shown by the aerobic composting is found to significantly reduce environmental odour (Zang et al., 2016).

Compost evaluation is important especially for eventual application. Compost analysis consists of physical, chemical, and biological analysis. Specifically, the criteria of physical analysis include temperature, colour, and odour, while the criteria of chemical analysis include carbon to nitrogen ratio, pH, and electrical conductivity (Wichuk and McCartney, 2013). The criteria of biological analysis include phytotoxicity tests, such as germination and pathogenic microorganisms (Cesaro et al., 2015). Overall, these analyses emphasise the importance of a stable compost for the end-user.

The use of good and quality compost is advantageous to soil health. Essentially, compost consists of humus, which is a good agent for soil amelioration (Fischer and Glaser, 2012). It also contributes to effective nutrient supply to plants (Hernández et al., 2016), especially crop macronutrient nutrition (Kalantari et al., 2010). Furthermore, compost has also been shown to improve plant growth upon its use as a growing medium for plant seedlings (Carlile et al., 2015; Rosenani et al., 2016) and as the soil mixture for advanced planting materials. Overall, a linkage is present between MARDI in-house producing compost and the potential of its application.

Although a composting project is viable in terms of production capacity, support is required for its potential use from the economic perspective. A study by Bong et al. (2017) in Iskandar City, Malaysia suggested that composting can be economically feasible with higher production volume. Similarly, composting can also be profitable should it be performed in a centralised plant (Zulkepli et al., 2017). Besides, a medium-scale composting has a higher potential to be financially feasible (Sabki et al., 2018). Taking this into consideration, a passive windrow aerated composting may be one of the low-cost processing options (Couth and Trois, 2012).

This article discusses the process of yard waste inventory and composting at MARDI headquarters and the evaluation involved to determine the quantitative and qualitative aspects of initial raw materials and compost products. The economic analysis and the prospect of the implementation of this technique on a larger scale are also highlighted.

## 2 Methodology

### 2.1 Waste collection, inventory process, and designing of the compost processing facility

The area selected for this study was the headquarters of MARDI (2° 59′ 51.4374″ N, 101° 41′ 26.227″ E), which is situated in Serdang, Selangor, Malaysia. The inventory of yard waste was conducted for seven months from the 1^st^ week of June 2017 until the 3^rd^ week of December 2017. Specific collection points were determined for the yard waste, which was detected using GPS handheld device (Garmin Monterra®) and drawn in a map through software Arc-GIS version 10.1. The wastes were collected and managed by a team of personnel from the Asset Management Center (AM). Overall, the procedures of the study comprised the transport of the wastes to the compost processing facility, which was performed two times weekly under the supervision of researchers from the Agrobiodiversity and Environment Research Center (BE) and the Soil and Fertilizer Research Center (SF). Both of these research centres were under MARDI. The compost processing facility was designed to consists of four main areas, namely; i) Area A (waste loading area), ii) Area B (weighing area), iii) Area C (composting area), and iv) Area D (post composting and storage area). The inventory of the fresh and dry weight was made upon the arrival of the wastes at the facility, which was followed by the process of composting yard waste.

### 2.2 Composting formulation and process

Upon the completion of the inventory, the wastes were mixed with the livestock manure and other wastes to achieve the ideal initial C/N ratio and a proper composting. Raw samples were analysed through chemical analysis to determine the initial C/N, followed by continuous supervision on the progress when the composting was performed. The technique implemented for all the heaps was the aerated (turned) aerobic composting, which involved a total complete cycle ranging from 60 to 90 days. The final products were evaluated on the physical and the chemical characteristics of the waste upon the completion of composting.

### 2.3 Physico-chemical analysis

The physical analysis involved the gravimetric identification of fresh weight and dry weight analysis. The weight of the bulk wastes obtained from the yard collection was measured using the CARIX-3015 electronic fully load cell floor scale. Samples were collected and divided into three categories, namely dry leaves, green leaves, and grass cutting. This was followed by moisture analysis, which was performed in triplicate using the MX-50 (AnD) moisture analyser to determine the dry weight. Then, the gravimetric test, which consisted of the final compost product, was conducted. In this phase, the samples were analysed in terms of yield and dry weight composition. Throughout the composting cycle, the temperature of the compost heaps was measured using a portable thermocouple (8 × 1000 mm) from the beginning until the end of composting.

The chemical analysis implemented a similar method used in the physical analysis of the triplicate samples. This analysis determined the pH, EC, ash content, and the percentage of organic C and kjedahl N, which values were used to determine the C/N ratio. The analysis of pH and EC was performed using the ratio of solid compost to water, which was 1:10 with Eutech PC700 (Eutech Instruments). The analysis of organic C was determined using the method by Walkley and Black (1934), while Kjedahl N was analysed using the method of Bradstreet (1954). The identification of C/N in this study adopted a similar method from Huang et al. (2004), where C was evaluated using the total organic C, while N adopted the method of the Kjedahl analysis. Additionally, the ash content (percentage) was determined through the incineration in a muffle furnace at 500 °C for 3 h, which was performed based on the method by Jakobsen (1995).

### 2.4 Germination test and microbiological analysis

Samples were analysed in triplicate, each weighing 20 g for the germination test. The extraction was performed with the ratio of solid compost to water at 2:1 (California Compost Quality Council, 2001). The extract along with the samples was filtered by Whatman filter paper (No. 1). The aliquots were used for the trial of the germination test, which involved the observation of germination of 20 *Vigna radiata* seeds (green bean) at 24, 48, and 72 h and conducted on a 10 cm petri dish. The final results were evaluated within 72 h, while the percentage of seed germination was calculated based on the number of germinated seeds over the total number of seeds (Luo et al., 2018).

The presence of *Salmonella* was evaluated using the Xylose Lysine Desoxycholate (XLD) agar plates. The extract was incubated for 24 h at 35 °C to determine the pathogenic activity (Pandey et al., 2016). The evaluation of the *Escherichia Coli* was carried out using the Eosin Methylene Blue (EMB) agar, while the characteristics of the bacteria were identified through green metallic sheen on the plates (Onifade et al., 2015).

### 2.5 Economic analysis

The cost and revenue from compost production were analysed for the feasibility of the project. The calculation was performed on economic indicators, including net and gross returns, overall price, and values of production in the analysis (Salehi et al., 2014), followed by the division of the total cost into operational (variable) and fixed (capital) costs. The values for the fixed cost were normalised based on the utilisation period (Bong et al., 2017). The analysis of the ratio of benefit to cost was also included to determine the overall viability of the project (Moqsud et al., 2011).

### 2.6 Descriptive statistical analysis

Microsoft Office Excel (MS Excel) and MINITAB Version 17 were used to perform statistical analysis, which involved the calculation of the mean values, minimum and maximum values, coefficients of variation (CV), and standard deviations (SD) for the characteristics of the raw material and the compost product. Although more emphasis was placed on the final results to evaluate the uniformity of the data, inventory sample, including the total initial weight and compost yield, was not statistically analysed as the samples were non-replicable. Besides, this sample was not applied in the germination and microbiological results as most of the values were either in the numerical form of 100% or non-detectable (N.D).

## 3 Results and discussion

### 3.1 Inventory of waste and design of compost processing facility

**Figure 1:**
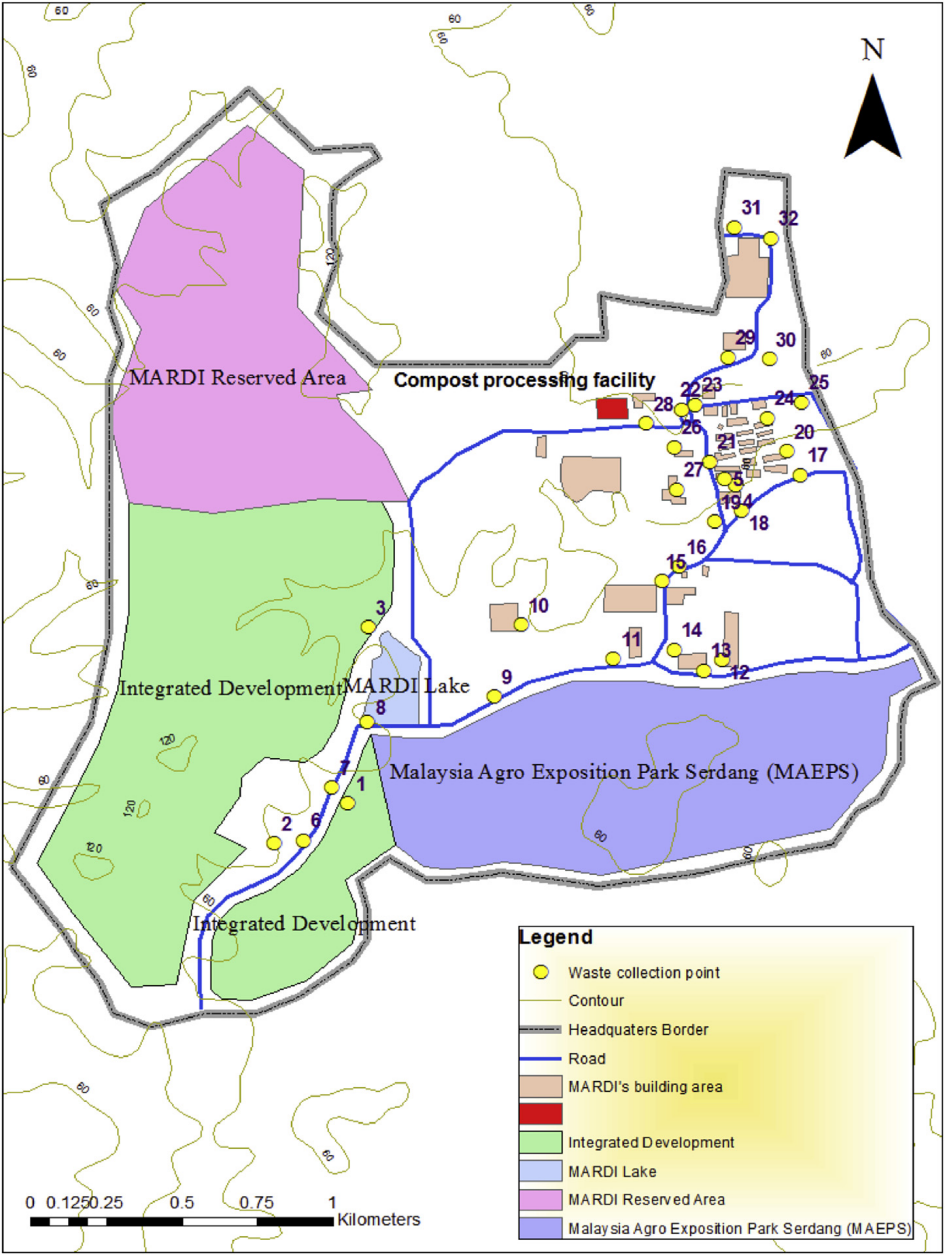
Location of compost processing centre and waste collections points in MARDI, Serdang, Selangor, Malaysia.

The total yard waste generated from June to December 2017 amounted to 16.75 tonnes with an average of 0.60 tonnes evaluated per week on the fresh weight (f.w.) basis. Figure 1 presents the waste collection points. The landscape wastes collected in plastic bags (89 cm × 102 cm) were arranged in small piles before being transferred to the compost processing facility, which was located within the vicinity of the institute, as seen in Figure 1. Table 1 illustrates the area required for each of the process taken place at the composting facility. Furthermore, the designated area highlighted the importance of proper design and spaces for the composting process (Shahudin et al., 2013; Vigneswaran et al., 2016). The loading of waste in Area A at the facility involved the evaluation of the amount of waste based on the fresh weight in Area B (refer to Table 1), followed by an analysis of the dry weight composition at the laboratory.

**Table 1:** Required workspace for the composting facility.

No	Type	Required area
**Area A (waste loading area)**		

1	The loading area for yard waste	6 m × 3 m
2	Loading area for livestock (nitrogen) waste	6 m × 3 m

**Area B (weighing area)**		

3	Weighing area (including fully-loaded floor scale)	6 m × 2.5 m

**Area C (composting area)**		

4	Composting area A	18 m × 9 m
5	Composting area B	14 m × 6 m

**Area D (post-composting and storage area)**		

6	Temporary storage (in jumbo bags)	6 m × 6 m
7	Compost refining/shredding	6 m × 3 m
8	Compost packaging and storage (small packages)	6 m × 3 m

**Figure 2:**
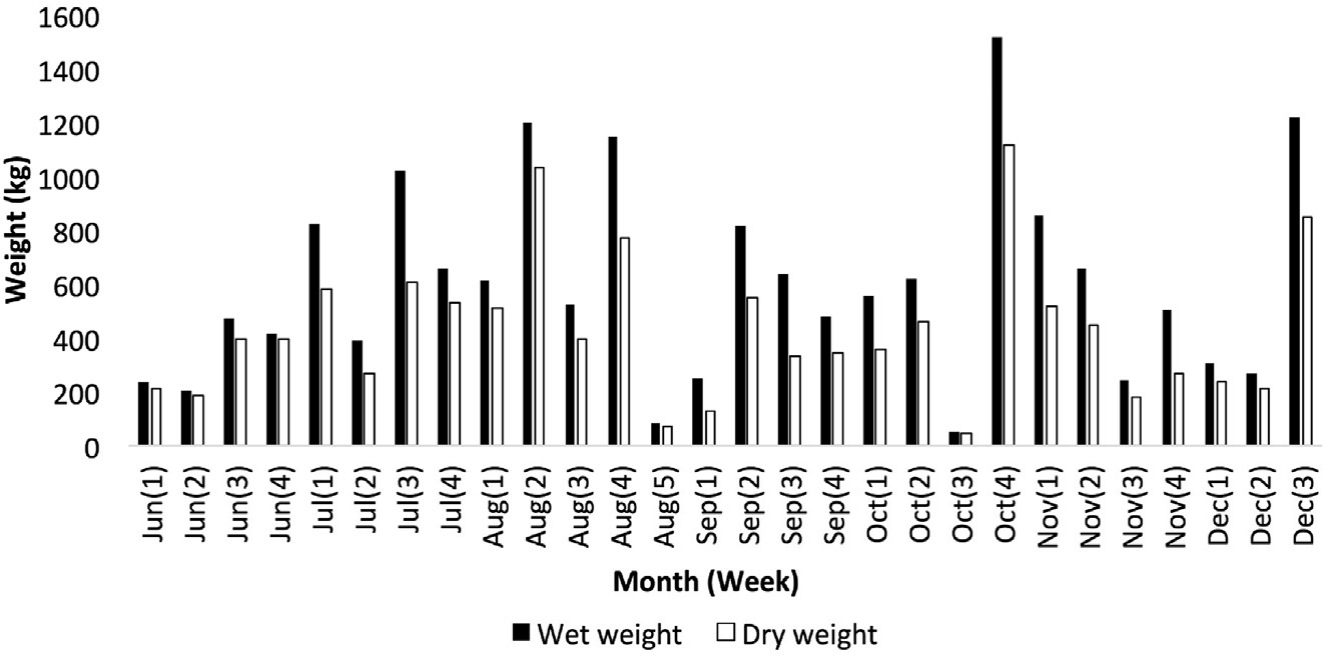
Total yard waste (wet and dry weight composition) generated from MARDI headquarters.

Figure 2 illustrates the amount of waste collected and its dry weight composition every week on the d.w. basis. Evidently, waste supplies were not evenly distributed throughout the seven-month duration. This phenomenon was due to the dependency of the collection on the availability of waste and the routine activity of the collection personnel, which required other tasks besides waste management. In addition, the minimal waste collection in a particular week was constantly followed by a surge in the collection of the following weeks, specifically the first week of September, the third week of October, and second week of December. Figure 2 also demonstrates that August consists of five weeks on a technicality as the maximum number of weekdays occur for five consecutive weeks in this month.

**Figure 3:**
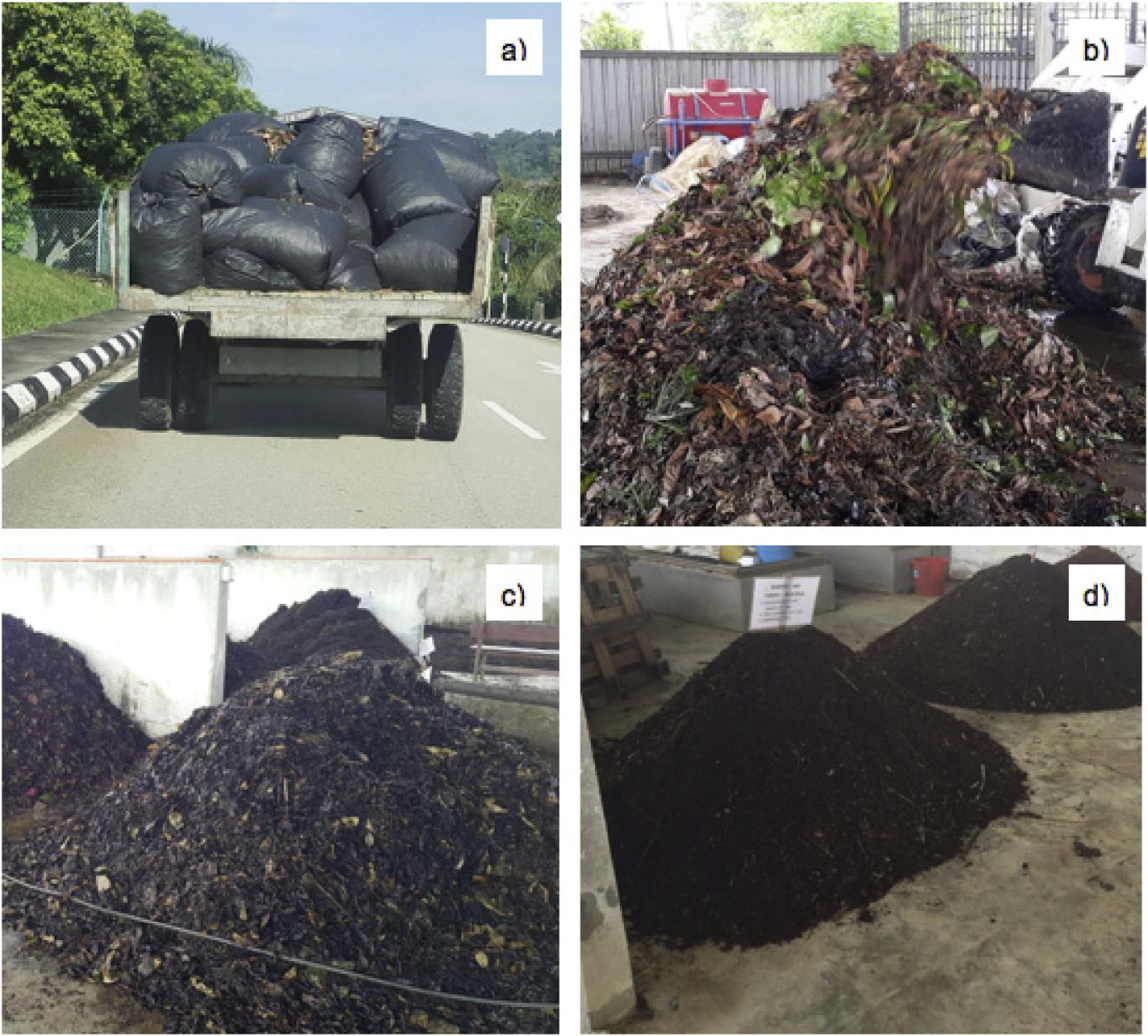
Yard waste composting activities in MARDI, Serdang, Selangor, Malaysia. (a) Yard waste collected weekly in large plastic bags transported to the compost processing facility; (b) Mixture of yard waste with N sources (mostly livestock manure) at the beginning of composting; (c) Compost heap within 35 days; (d) Compost heap at the end of the process.

The percentage composition of yard waste was one of the most challenging elements to be accurately quantified due to the absence of the standardised or commonly accepted characterisation methodology concerning the MSW composition studies (Edjabou et al., 2015). As a result, the wastes were gravimetrically estimated in this study. However, the proportion of waste percentages should be performed through the expert's judgement. Moreover, segregation was an almost impossible process as the yard wastes were heterogeneously mixed and packaged in plastic bags before they were transported to the compost processing area (refer to Figure 3).

In this study, an expert's judgement was defined as individuals who work extensively in the inventory process, including technical field workers and officers. In the inventory process, discussions were carried out to determine the waste proportions for each of the received batches. This study implemented the visual estimate of the volume, which was similar to one of the approaches adopted in Burnley et al.’s (2007) study on the assessment of municipal waste (refer to Table 2). Following that, the estimated percentage of the volume was converted to dry weight values based on its proportions. The estimation characterised three types of yard waste for analysis, namely dry leaves, fresh green leaves, grass cuttings. Specifically, dry leaves accumulated to form a bulk of the wastes and was estimated to constitute 70%–100% of the waste composition. The observation and estimation was justified based on the actual scenario of the natural littering of the dry leaves. It was a continuous process of the management personnel to collect the waste to maintain the cleanliness of the landscape area in MARDI.

**Table 2:** Compost mixtures (d.w. basis) for yard waste and additional N sources, which include the estimated percentage of yard waste composition.

Heap	Yard waste			Additional N sources (livestock and plant-based kg d.w.)	Number of inventory batch (yard waste)	The estimated fresh weight percentage volume of yard waste (Dried leaves: Green leaves: Grass cuttings)
	Dried leaves (kg d.w.)	Green leaves (kg d.w.)	Grass cuttings (kg d.w.)			
1	391.3	-	-	360.6	2	100:0:0/100:0:0
2	383.2	11.9	-	287.9	1	90:10:0
3	267.1	34.8	-	304.5	1	80:20:0
4	394.1	183.5	-	516.7	2	50:50:0/70:30:0
5	645.5	39.6	188.8	442.6	3	70:30:0/60:0:40/60:0:40
6	415.1	-	112.6	403.0	1	80:0:20
7	469.2	-	39.9	187.5	2	95:0:5/90:0:10
8	953.8	-	78.8	587.5	1	90:0:10
9	454.3	2.6	48.6	307.3	3	80:0:20/50:50:50/90:10:0
10	524.1	137.1	-	220.0	1	70:30:0
11	493.2	-	63.5	166.0	4	90:0:10/90:0:10/90:0:10/90:0:10
12	160.1	-	36.0	105.9	1	90:0:10
13	310.6	22.5	-	106.7	1	80:20:0
14	310.3	36.2	-	94.5	1	80:20:0
15	301.9	-	56.2	121.3	1	70:0:30
16	380.2	21.7	56.5	182.8	2	80:0:20/80:20:0
17	315.4	20.8	57.0	135.7	2	100:0:0/70:15:15
18	648.2	67.9	54.5	114.1	2	80:20:0/70:15:15
19	454.8	61.8	-	263.8	1	80:20:0
20	567.9	37.8	20.6	449.5	4	80:0:20/80:20:0/90:0:10/80:10:10
21	249.5	13.2	2.9	486.3	2	85:0:15/80:20:0
22	176.7	61.5	-	223.4	2	70:30:0/70:30:0
23	776.8	87.6	235.0	561.4	4	70:20:10/80:20:0/50:20:30/85:15:0

Grass cutting was the second abundant sources of yard waste. It was produced in a selected period (seasonal), with lower percentages compared to dry leaves. In contrast, green leaves constituted the minimum portion of the waste and were collected in the pruning process. In this case, the solid pruning waste was managed and separated using other techniques as the waste was not suitable for composting compared to the leafy parts. The combination of green leaves and grass cuttings constituted 20%–30% of the waste. Notably, analysis of the total dry weight of the waste in Table 2 contributed to important information of the initial formulation of the compost. It also provided useful information compared to the completed (matured) compost, which will be presented in the next section.

### 3.2 Initial composting mixture and formulation

A total of 23 heaps of compost was produced, with the first heap starting in early June 2017 and the final heap (no. 23) starting in late December 2017. The composting of the final heap completed approximately three months later. The production of the compost followed the aerobic composting method, in which raw materials were initially mixed and the process of turning took place every three days to ensure that the compost was homogeneously mixed. The importance of turning also influenced other parameters, such as temperature, carbon, and mass loss (Tiquia et al., 2000). Provided that the period of composting and the compost quality are important, the correct technique of composting was constantly emphasised.

The maximum size of the compost heap at the first stage of composting was 3.5 m (W) x 3 m (L) x 2 m (H). In respect of composting areas in Table 1, composting area A could accommodate the maximum of six heaps of compost, while composting areas B could accommodate another four heaps of the compost. As a result, an overall maximum of 10 heaps was accommodated. In this arrangement, the movement area for the skid steer loader machine was considered at approximately 3 m within the main alley for areas A and B. This steer loader was used for compost turning purpose. Throughout the composting period, the maximum of 10 heaps of compost was obtained on the 22^nd^ week, which was at the end of October 2017. However, five of the heaps were more than six weeks and was reduced by approximately 50% to the size of 2 m (H) x 1.5 m (L) x 1 m (H). Nevertheless, the occupancy rates ranged from eight to nine heaps in other points of time.

One of the main considerations in conducting a composting process was the C/N ratio (Michel et al., 1996), which was one of the mandatory requirements for compost analysis (California Compost Quality Council, 2001). Essentially, an appropriate formulation of compost is critical to ensure that the initial C/N is neither too high nor too low and ideally within the range from 25:1 to 30:1 (Pace et al., 1995). Provided that the yard waste was heterogeneously mixed, the initial C/N was determined based on the estimated weight composition of waste and its pre-analysed nutrient composition of the raw materials (refer to Table 3). The values of the C/N ratio, which were weight composition, were calculated based on the formula by Richard and Trautmann (1996).

**Table 3:** Characteristics of raw materials used for composting (Values = mean ± standard deviation at n = 3).

Raw materials	Moisture%	C%	N%	C/N	Ash%	pH	Electrical Conductivity (mS/cm)
Goat dung (organic)	28.09 ± 2.25	25.25 ± 0.93	1.04 ± 0.12	24.28	21.43 ± 4.74	7.71 ± 0.09	7.71 ± 0.02
Goat dung (conventional)	41.87 ± 6.86	27.57 ± 0.75	1.63 ± 0.33	16.91	12.47 ± 2.55	8.17 ± 0.07	9.60 ± 1.02
Horse dung	45.58 ± 1.94	27.07 ± 1.53	1.42 ± 0.09	19.06	7.71 ± 5.07	7.01 ± 0.02	8.51 ± 0.08
Dry leaves	10.75 ± 1.29	33.27 ± 1.07	0.76 ± 0.09	43.78	4.74 ± 0.63	5.12 ± 0.16	0.82 ± 0.13
*Lagerstroemia* sp	12.08 ± 0.19	21.92 ± 2.08	0.62 ± 0.12	35.35	12.08 ± 0.19	7.15 ± 1.75	0.82 ± 0.11
*Ficus benjamina*	24.63 ± 2.16	34.42 ± 0.67	2.32 ± 0.14	14.84	24.63 ± 2.16	7.04 ± 0.05	0.99 ± 0.05
*Artocarpus* sp	11.41 ± 0.63	35.77 ± 2.51	0.68 ± 0.16	52.6	11.41 ± 0.63	5.09 ± 0.09	0.77 ± 0.04
*Cinnamomum* sp	14.55 ± 0.65	42.50 ± 2.85	0.90 ± 0.15	47.22	14.55 ± 0.65	5.42 ± 0.13	0.30 ± 0.05
Mahogany	10.99 ± 0.20	42.88 ± 3.18	1.54 ± 0.26	27.84	10.99 ± 0.20	5.18 ± 0.09	0.76 ± 0.03
*Acacia* spp	10.23 ± 0.28	45.58 ± 2.31	1.24 ± 0.03	36.76	10.23 ± 0.28	5.00 ± 0.08	0.67 ± 0.16
Green leaves	50.60 ± 4.11	33.86 ± 3.65	1.16 ± 0.23	29.19	5.64 ± 0.34	4.71 ± 0.17	0.85 ± 0.20
Grass cutting	38.85 ± 7.74	29.06 ± 0.43	1.15 ± 0.07	25.27	3.34 ± 0.61	6.02 ± 0.08	1.05 ± 0.04
Rice mill waste	11.83 ± 0.13	29.21 ± 0.45	0.94 ± 0.10	31.07	19.63 ± 0.37	7.28 ± 0.14	0.85 ± 0.03
Vegetable waste (cabbage)	82.05 ± 0.96	34.32 ± 2.58	3.29 ± 0.15	10.44	20.62 ± 0.06	5.82 ± 0.33	0.21 ± 0.01

Based on the C/N for every raw material presented Table 3, it was found that dry leaves had high C/N ratio, which led to their integration with other sources to lower the values. Six major species of landscape plants, which were collected within the MARDI area and became the sources of the dry leaves, consisted *Lagerstroemia* sp, *Ficus benjamina*, *Artocarpus* sp, *Cinnamomum* sp, *Mahogany*, and *Acacia* spp (refer to Table 3). Despite the number of species for the major plants, most of the dry leaves were identified as *Lagerstroemia* sp, *Artocarpus* sp, and *Mahogany*. The percentage of C ranged from 21.92% to 45.58%, while the percentage of N ranged from 0.62% to 2.32%. Additionally, the average percentages of C and N, which were adopted to represent the dry leaves, amounted to 33.27% and 0.76%, respectively. Although these values were then extracted from the analysis of the leave mixture, they were comparable and representative of the total major composition of the dry leaves.

**Figure 4:**
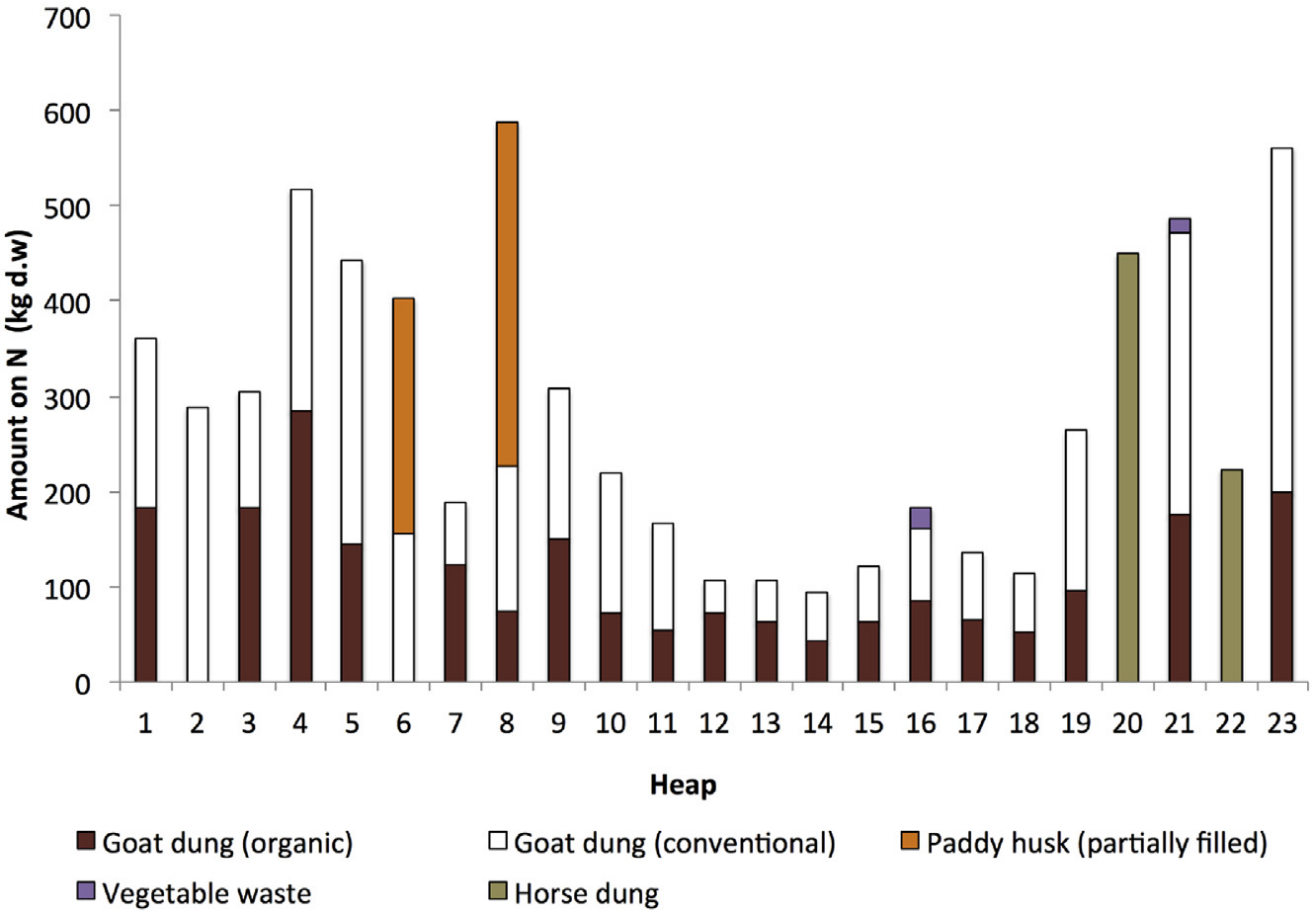
Amount of N sources added to the compost heap (dry weight basis).

Based on Table 3, materials with good N sources were goat dung, horse dung, green leaves, and grass cuttings. Although the C/N ratio for these sources exceeded 15:1, it could be a good mix and suitable additional N source for the dry leaves. Although an ideal initial C/N ratio could not be achieved due to the limitation of the N sources within the study area, the use of the C/N formulation ensured that adequate N sources were added to the yard waste before the composting process. The analysis also indicated that the C/N ratio of the initial composts ranged from 24.8 to 41.8 (refer to Figure 6). The total amount of the N sources added to the compost heaps is illustrated in Table 2, while its fraction composition on the d.w. the basis is illustrated in Figure 4.

### 3.3 Composting process and physicochemical analysis

#### 3.3.1 Temperature profile of the composting heaps

**Figure 5:**
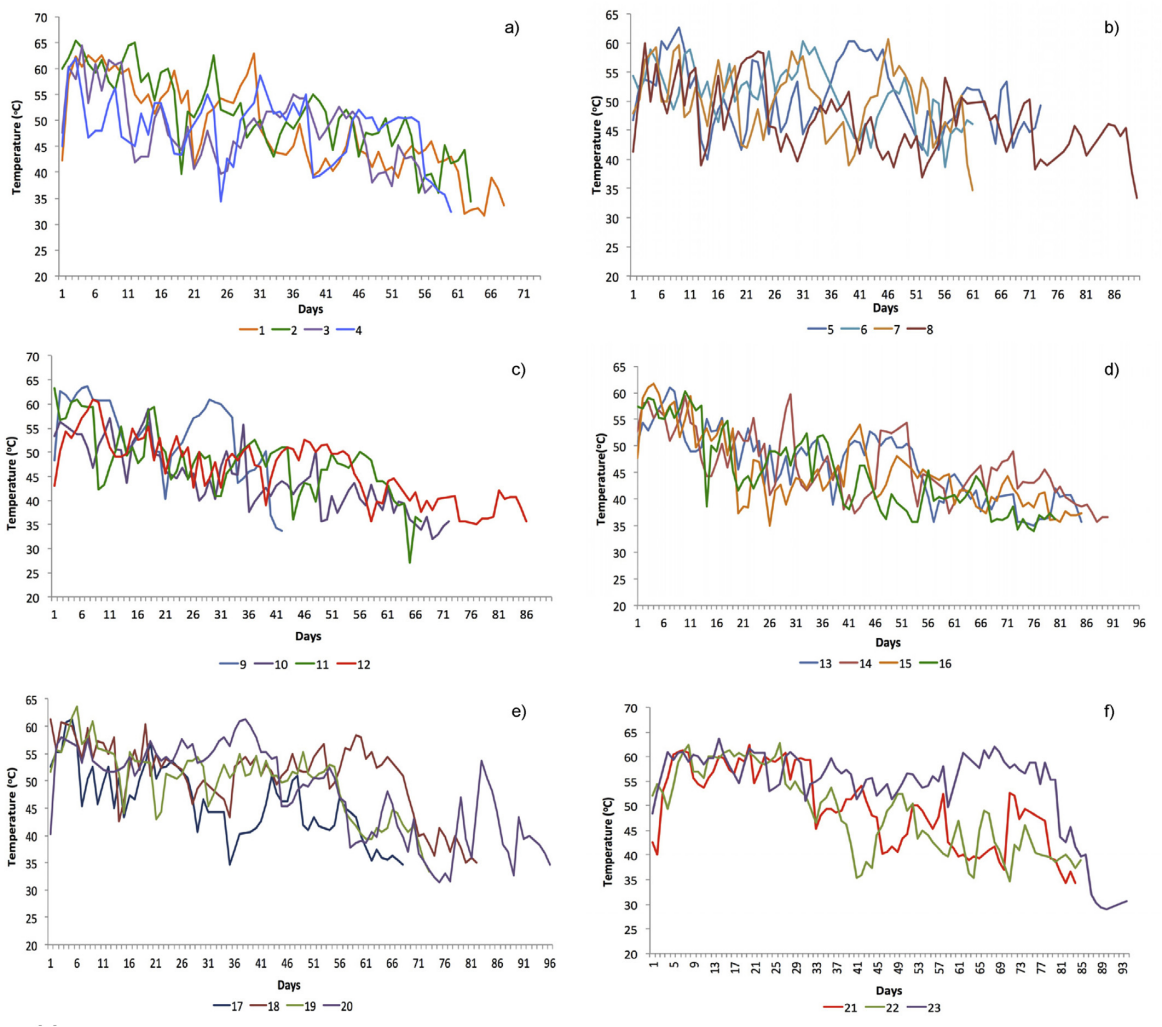
The compost heap temperature profiles from code 1 until code 23 (a–f) produced from the waste generated from June to December 2017.

Temperature is one of the important parameters in composting, and it determines the stages and progress of the compost piles (Trautmann et al., 1996). The first step in the evaluation of the on-going composting depends on the temperature profile and the observation on the compost features. Furthermore, the optimal temperature for thermophilic ranges from 55 °C to 60 °C (Golueke, 1991a), and the active phase of composting refers to the increase in the pile to thermophilic temperatures (Cooperband, 2002). Based on the temperature profiles obtained in this study, similar trends of thermophilic phase were observed at 60 °C at the beginning of composting, followed by the reduction in the heap temperature (refer to Figure 5). Fluctuations were found in the compost temperature through the process as a result of turning activities, and this process resulted in a temporary decrease in the temperature of compost heaps before returning to the initial temperature.

It was found that the temperature trends for the yard waste composting were relatively different from other homogenous sources, such as rice straw (Mohammad Hariz et al., 2013), barley straw (Kulikowska and Sindrewicz, 2018), and cassava peels (Mary et al., 2014). It was indicated from the trends observed in this study that a longer thermophilic phase was present from the temperature, while the cooling phase was stretched from the thermophilic phase for several days. This trend was also observed in the study by Wong et al. (2001) on the combination of leaves and soybean residues. The study regarding the use of leaves or yard waste as the input for the composting suggested that longer thermophilic and cooling period was previously present. Similarly, the study by Hartz and Giannini (1998), which evaluated the performances of the composted yard waste, also showed similar temperature variations, in which a prolonged fluctuation of over 50 °C was observed from the temperature.

The decreased temperature of most of the heaps was observed in Figure 5. Meanwhile, Tiquia et al. (1996) identified the patterns of heap temperature, which decreased between 30 °C and 40 °C towards the end of composting (maturing stage), where the curing process occurred and compost reached its stability and maturity. Furthermore, Golueke (1991b) elaborated on the determination of the finished compost from the reduced temperature, indicating a failure of the materials to reheat after a turning process. The compost at a consistently low temperature indicated the end of composting (Kuba et al., 2008). Provided that the compost could be stored for a certain duration of time before application, it was safe to be placed on agricultural soils. Therefore, the practices in this study considered the composting process had reached towards stabilisation when the temperature was below 40 °C and failed to be reheated on two to three days after the turning process.

The cut-off temperature of below 40 °C indicated that the composts were complete, which was followed by the process of temporary drying and storage in large jumbo bags. Notably, the decision on the cut-off temperature was based on the observations in the previous studies. In addition to the trends of temperature variation in yard waste composting by Hartz and Giannini (1998) and Wong et al. (2001), several studies indicated that the completion stage of compost was reached when the temperature of the piles ranged from 35 °C to 40 °C. This finding was also developed in the studies by Awasthi et al. (2015), Van Fan et al. (2016), Zhang et al. (2018), and Tibu et al. (2019). Therefore, it was followed in this study to perform the drying and storage processes.

The stabilisation of the average composting took place for 60–80 days. It was observed that an early stabilisation took place on heap no. 9 on the 40^th^ day, as shown in Figure 5c. Following that, an analysis of C/N was performed to determine the degree of the finished compost. As a result, samples were found to have the same attributes in all the heaps, which were similar to the attributes of the completed and finished compost, including dark (almost black) in colour and an earthy smell (Baharuddin et al., 2009).

Anomalies and prolonged high-temperature levels were observed on certain heaps in this study, as seen from compost 5, 8, 20, and 23 (refer to Figure 5). In this case, the compost characterisations should be determined in the physical and chemical aspects through the in-situ and ex-situ approaches. Provided if the compost was blackish, the samples would be obtained to determine the final C/N ratio to confirm the completion of the composting process. This phenomenon was observed in heaps no. 5, while the data collection regarding the temperature profiles was extended for heaps 8, 20 and 23. Therefore, it was indicated that the composting process took nearly 90 days (three months) to reach a stabilised temperature below 40 °C.

Spatial variations may play certain roles in the prolonged high temperatures of the heaps on the degree of composting. A study by Tiquia and Tam (2000) on forced aerated compost illustrated that temperature was related to different chemical and biological parameters. Although turning was performed frequently to ensure homogeneity, the decomposition process might differ from one heap to another. Therefore, certain fluctuations of core heap temperatures were more evident compared to the others, indicating that an active decomposition process still took place and increased the heap temperature.

#### 3.3.2 C/N ratio and nutrient balances of the finished compost

**Figure 6:**
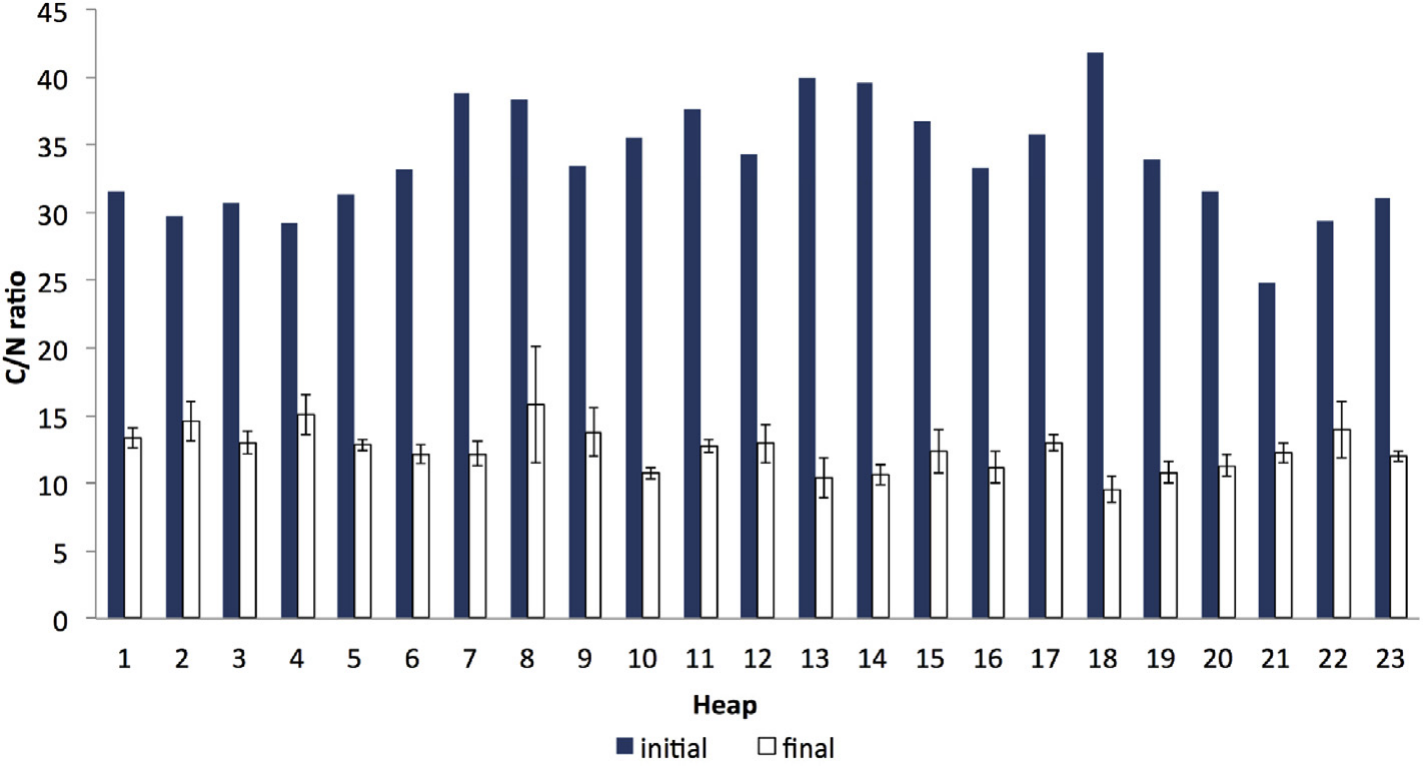
C/N ratio between initial and final compost (initial C/N was estimated based on the dry weight data). The bars on the final compost indicate standard deviation of the mean (n = 3).

Mature compost is a stable material with a slow biological activity (Fourti et al., 2013), while an immature compost may inhibit plant growth and induce anaerobic conditions when applied to the soil (Inbar et al., 1993; Mathur et al., 1993). It was indicated from the results that the final C/N ratios of all the composts ranged from 10:1 to 15:1 (refer to Figure 6). Furthermore, the sample from heap no. 9, which was reported in the previous sub-section to exhibit early temperature stability, also showed a final C/N ratio of 14:1 (refer to Figure 6). The overall standard deviation (SD) was measured at 2.054, while the coefficient of variation (CV) was found to amount to 16.3% and highlighted an acceptable range for the results.

Notably, a high C/N ratio may immobilise the nitrogen in plants instead of mineralising it (Chaves et al., 2005). Therefore, the evaluation of the C/N of the final compost is essential to ensure that its eventual utilisation contributes to positive results. Accordingly, a set of C/N criteria for an ideal compost was taken into account in several studies. Specifically, Hirai et al. (1983) and Hachicha et al. (2012) suggested that a matured compost ratio of C/N was lower than 20:1. Similar trends were also found in another study by Wei et al. (2014), while Bernal et al. (1998) suggested a preferable C/N ratio of 15:1 or lower. These criteria indicated that the results of this study were comparable to another result, and all the composts achieved the completion stage.

**Figure 7:**
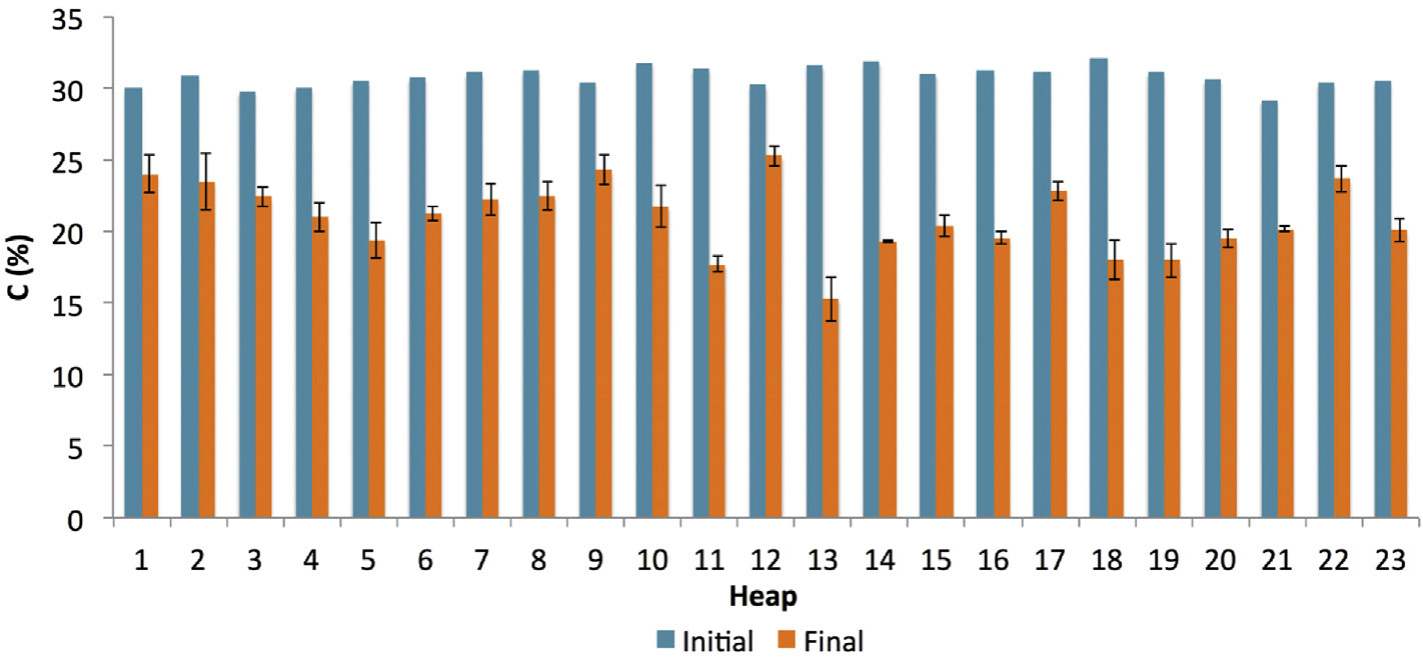
Percentages of C between the initial and final compost (initial C was estimated based on the dry weight data). The bars on the final compost indicate standard deviation of the mean (n = 3).

The remaining C contents in the final compost after the composting process could contribute to positive impacts on soil (Figure 7). Despite the presence of several percentages of labile carbon in the constituents, a part of the compost may be converted to humus, which forms a stabilised sequestered carbon (Whitehead and Tinsley, 1963). Furthermore, Hermann et al. (2011) highlighted that 23% of humus made up from the C contents in the compost reached its stability, which lasted for over 100 years. Therefore, various advantages of humus in soil may be attributed to the use of compost, such as improvement in nutrient uptake (Solaiman et al., 2019), soil organisms (Vasileva and Kostov, 2015), and aeration to soil (Leu, 2007).

**Figure 8:**
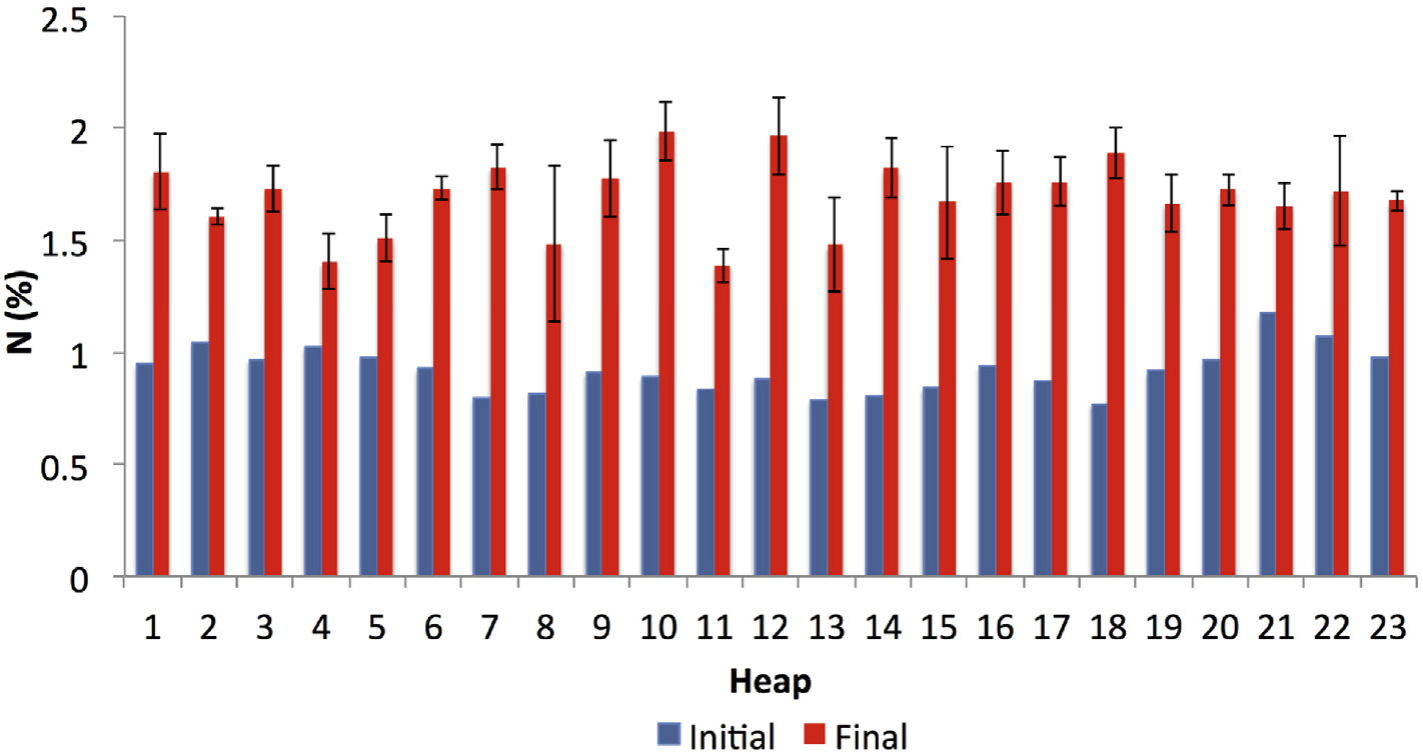
The percentages of N between the initial and final compost (initial N was estimated based on the dry weight data). The bars on the final compost indicate the standard deviation of the mean (n = 3).

Figure 8 illustrates the increased percentages of N in the final compost, which could be partly attributed to the loss of carbon substrate through CO_2_ despite the possible occurrence of minimal losses of N from volatilisation (Guo et al., 2012; Rasapoor et al., 2009). The acquired higher percentages of N in the compost would serve as important nutrients for crops (Maeda et al., 2011). The process of nitrification taken place in the nutrients would lead to the formation of N into an available form (Cáceres et al., 2018).

#### 3.3.3 Compost yield

**Figure 9:**
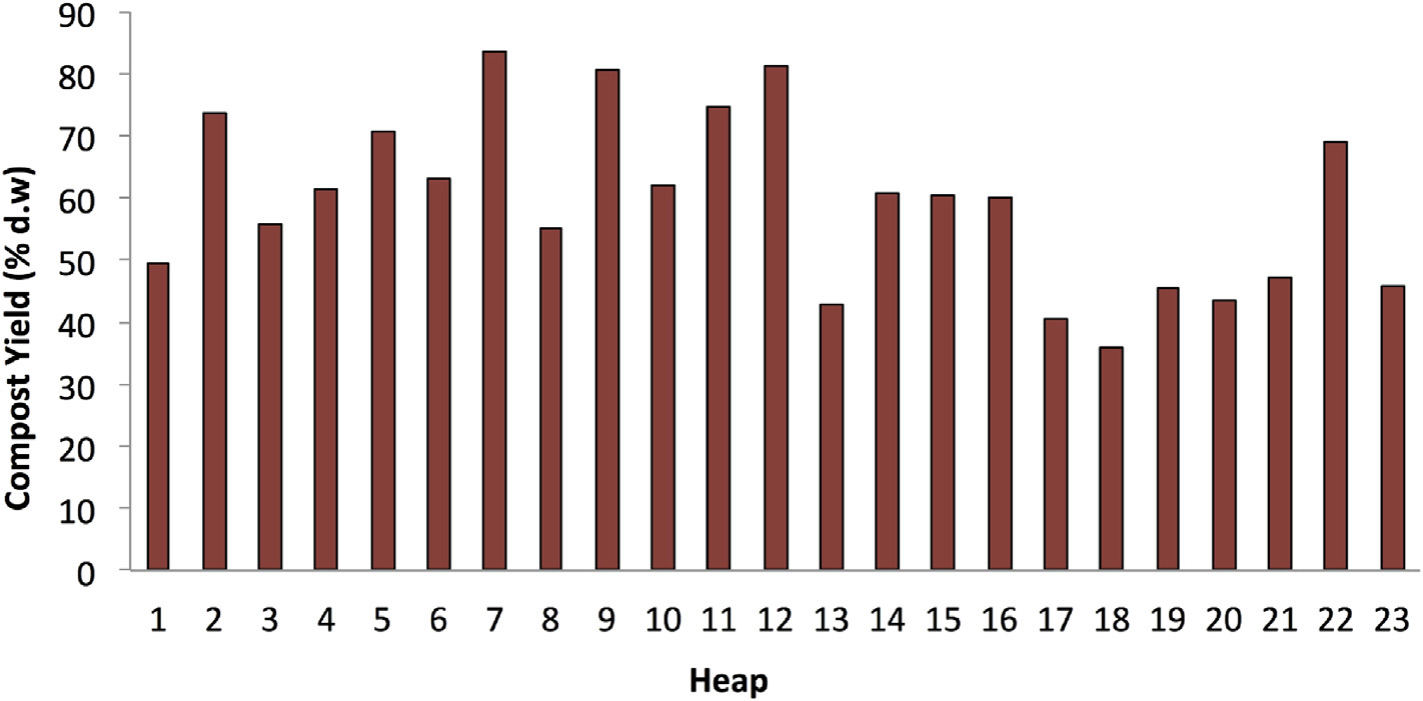
Percentage of compost yield (dry weight basis).

The compost yields mostly ranged from 40% to 70%. Accordingly, only six out of 23 heaps exhibited a compost yield of over 70%, while one heap had a compost yield of lower than 40%. The study by Adhikari et al. (2009) identified a mass reduction of approximately 50% for the composting of food wastes with chopped hay and wood-shaving, while Pan et al. (2012) observed a weight loss of nearly 30% or approximately 70% of compost yield. Comparatively, it was comparable to see that the average loss for most of the heaps reached the value of 50% (refer to Figure 9).

#### 3.3.4 Ash content

**Figure 10:**
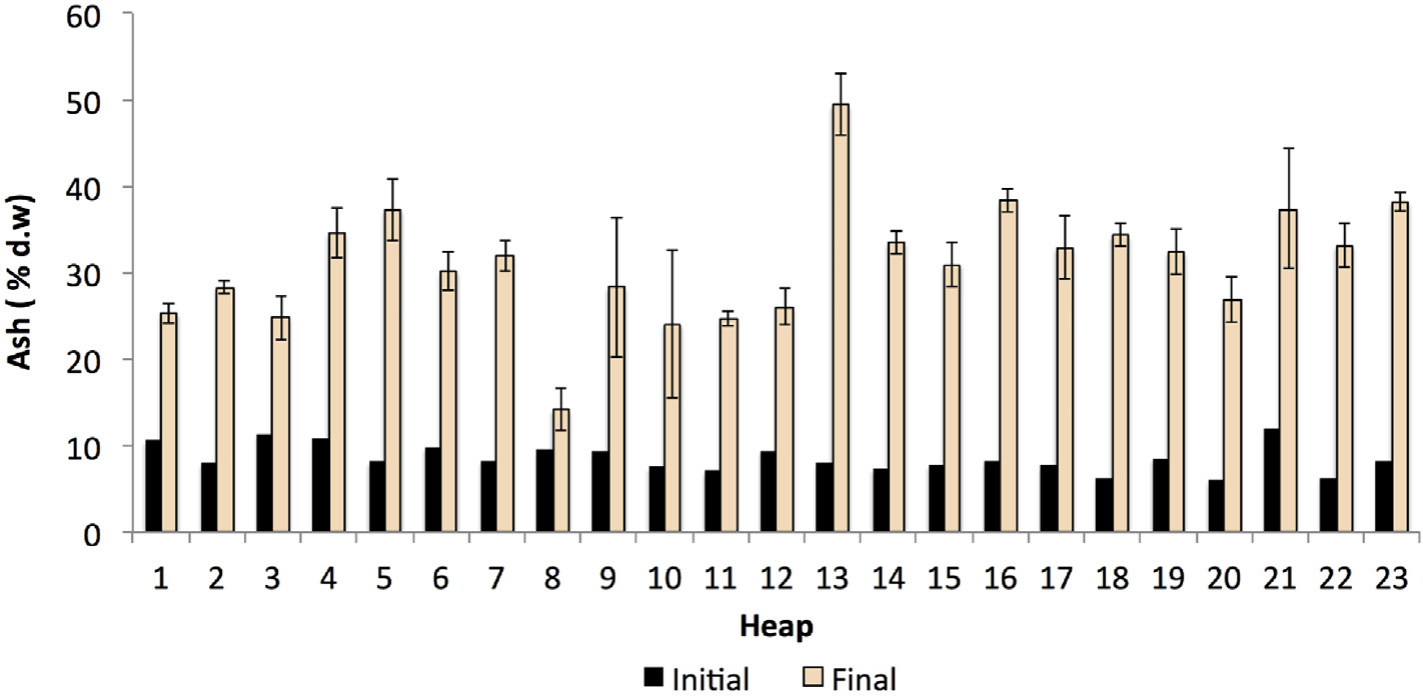
Ash content (%) in the initial and final compost (initial ash percentage was estimated based on the dry weight data). The bars on the final compost indicate standard deviation of means (n = 3).

As seen in Table 3, lower ash content was observed in the raw materials, with an exception for rice mill waste, vegetable waste (cabbage), and goat dung (from the organic farm). The data presented in Table 2, Table 3, and Figure 4 were used for the estimation of the initial ash content of the compost. As a result, it was found that the initial ash contents for all heaps ranged from 6.25% to 12.01% (Figure 10), while the final ash contents of the compost ranged from 24.70% to 49.37% in average, with heap 8 as an exception as its ash contents were slightly below 20%. Nevertheless, the contents remained above the initial compost mixture (refer to Figure 10). Similarly, the overall SD for the ash content amounted to 7.407, while the CV amounted to 23.9%, which remained within an acceptable range of lower than 30%. Overall, uniformity was present in the results of the ash contents.

The final characteristics were compliant with the characteristics shown in the previous study, which indicated that although the ash content was initially lower in the raw materials of composting, the percentage of it increased at the end of the process (Hsu and Lo, 1999). This finding was comparable to other research by Haynes et al. (2015) and Wang et al. (2015), in which Waqas et al.’s (2017) study on the co-composting of food waste with biochar found that the ash content ranged from 35% to 45%, proving that higher ash content indicated efficient and high decomposition rate of organic materials.

#### 3.3.5 pH and EC

**Figure 11:**
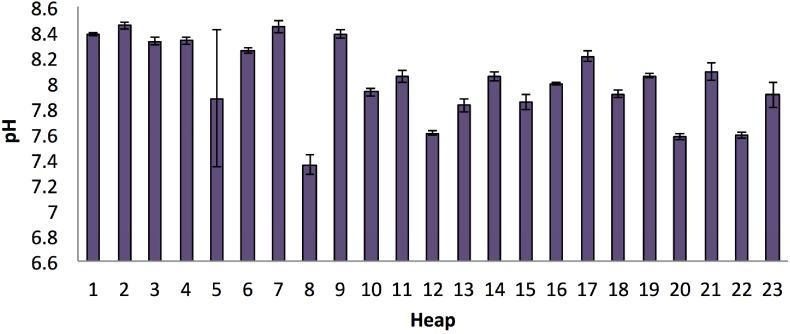
pH of the final compost product. The bars on the graph represent the standard deviation of means (n = 3).

The composts mostly exhibited alkaline characteristics, as shown in Figure 11. It was indicated from the results that the pH of the compost ranged from 7.35 to 8.45. While the overall SD amounted to 0.316, the overall CV was significantly lower by 3.9%. Furthermore, the pH of the raw materials was measured at an average of 5.12 for the dry leaves of the yard wastes (Table 3). The pH levels for goat dung from the two sources (organic and conventional) were 8.17 and 7.71, respectively, while the pH for horse dung was 7.01. These results indicated that the pH of the products transformed from slightly low to alkaline.

The matured composts were normally associated with the characteristics of alkaline pH (Grube et al., 2006) and had alkaline properties at the end of the composting process (Lin, 2008). Notably, alkaline properties are useful for a specific application as the properties improve soil condition by inhibiting the acidification process. This phenomenon may occur as a result of N fertilisation (Walker et al., 2004). Moreover, the increase in pH was associated with the production and introduction of hydroxide and basic cations in the soil (Mkhabela and Warman, 2005). The attributes of composts obtained from this study suggested a specific application of compost, particularly in soils or planting media with lower pH.

**Figure 12:**
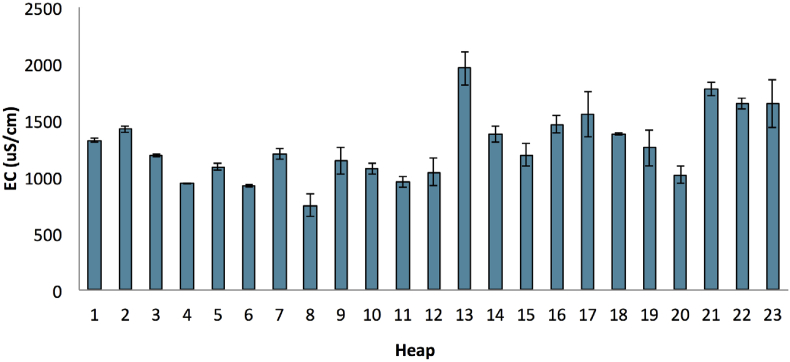
Electrical conductivity (EC) of the final compost product. The bars on the graph represent the standard deviation of means (n = 3).

The electrical conductivity (EC) of the compost ranged from 746.7 uS/cm to 1958.7 uS/cm (Figure 12). The overall SD amounted to 307.96, while the overall CV was within the precise value of 24.2%. Overall, provided that these values indicated that the composts had an acceptable range of soluble salts, they were suitable for a direct application as the media for plant seedlings (A&L Canada Laboratories Inc, 2004). It was indicated that the composts could be used for other purposes, such as soil amendments and transplantation of media combined with other sources. It could be seen in Table 3 that the values of EC for several raw materials were relatively high, indicating that a direct application of raw materials might potentially have negative effects on plant growth.

### 3.4 Seed germination and pathogenic test

The results of the seed germination test showed a highly successful germination rate of above 95% after 72 h (Table 4), which suggested that the composts were compatible with soil application. Notably, this test was crucial as one of the final evaluation processes performed on specific suitability of compost for utilisation. Being one of the tests recommended for maturity evaluation in the California Compost Quality Council (2001), it determined the toxicity of the compost (Luo et al., 2018). An excellent germination rate indicated that the compost could be used for soil mixture and conditioner.

**Table 4:** Seed germination rate, *Salmonella*, and *E. Coli* results of the compost.

Heap	Germination^a^ (%)	Salmonella^b^ (log 10)	Ecoli^b^ (log 10)
1	98.33	N.D	N.D
2	96.67	N.D	N.D
3	100	N.D	N.D
4	100	N.D	N.D
5	100	N.D	N.D
6	100	N.D	3.22
7	100	N.D	N.D
8	100	N.D	N.D
9	M.D	N.D	3.22
10	100	N.D	N.D
11	100	N.D	N.D
12	100	N.D	N.D
13	100	N.D	N.D
14	100	N.D	N.D
15	100	N.D	N.D
16	100	N.D	N.D
17	100	N.D	N.D
18	100	N.D	N.D
19	100	N.D	N.D
20	100	N.D	2.70
21	100	N.D	N.D
22	100	N.D	N.D
23	100	N.D	N.D

a M.D - Missing data.

b N.D - Non-detectable.

The use of *Vigna radiata* seeds in the germination test could be observed in several studies, including Gopinathan and Thirumurthy (2012), Fadzilah et al. (2017), and Rupani et al. (2017). Furthermore, the germination of *Vigna radiata* seeds was influenced by several factors, including salinity (Promila and Kumar, 2000), while acidic or low pH constituent led to adverse effects on it (Kannan and Upreti, 2008). Therefore, the composts with low salinity and slightly alkaline pH (Figure 11 and Figure 12) exhibited parallel results with the results from the study on seed germination (Table 4).

Another test besides the germination test was the pathogenic test, which focused on the pathogenic microorganisms. Notably, this test was important due to the impact of the presence of the microorganisms on the compost quality for eventual utilisation, particularly the sanitary and safety of humans through direct or indirect exposure. Besides, the British Standard Institution (2011) has established the PAS 100: 2011 standard requirement to determine *Escherichia Coli* and *Salmonella* sp. as one of the criteria for the pathogenic evaluation of compost quality. Subsequently, the upper limit for *Escherichia Coli* is 1000 CFU g^−1^ fresh mass. It could be seen in the results on *Escherichia Coli* in Table 4 that the upper limit for the three samples ranged from 2.70 to 3.22 log_10_ CFU g^−1^, which exceeded the detection limit of 1000 CFU g^−1^. However, other results presented non-detected microorganisms.

Based on the results in Table 4, the presence of *Salmonella* was not detectable. While similar trends of the absence of *Salmonella* was found, the minimum presence of *E. Coli* was found in other studies, including the study by Soobhany et al. (2017). Provided that contamination by pathogenic microorganisms normally occurs through the use of manures in composting, an evaluation was essential, especially as the N sources mostly originated from the livestock waste (Liu et al., 2013). Nevertheless, composting remained one of the ideal techniques to reduce and eliminate the risk of pathogenic microorganisms (Davis and Kendall, 2005).

### 3.5 Economic analysis and benefit to users

The economic viability of a composting project was subject to the potential market of the compost product and the cost-saving initiatives from the application of the compost. The use of compost could minimise the cost incurred by an institution or organisation to purchase the product for retail for landscaping purposes. Essentially, compost is the main ingredient for the growth media at various plant nurseries located within the institution. In MARDI, the compost was used mainly for soil amendment, particularly growing media and basal media, and in soil mixture for advanced planting materials. Notably, MARDI has been conducting the Green Campus programme since 2017 to plant more than 1000 new trees of various species. It also utilises the in-house compost, which follows its objective towards soil amendments.

The values of the compost could be represented through two scenarios. Specifically, Scenario 1 illustrates how the compost was sold in retail. A premium compost was sold at 1 kg per package with the price of it was estimated to be RM2.50 per kg. Notably, selling the compost as a premium compost might be among the options for the compost producer to develop a commercial venture from it. Meanwhile, Scenario 2 (refer to Table 5) illustrates the direct use of the compost by MARDI, which could save the annual allocation budget used to purchase the compost for landscaping purposes. Following the purchase of the compost in bulk by MARDI, the estimated value of the compost in Malaysian Ringgit (MYR) was RM1.50 per kg.

**Table 5:** Cost breakdown and revenue from compost production throughout the study.

Item	Scenario (values in MYR)		Remark
	1	2	
**(A) Operational Costs**			

1) Water consumption	55	55	Estimated based on commercial price.
2) Petrol for shredder	354	354	Approximate use of maximum 2 h for each heap.
3) Diesel for skip loader	1795	1795	Used for compost turning, weighing and storage.
4) Maintenance of skip loader (mini tractor).	1200	1200	Cost of service/maintenance.
5) Maintenance of shredder	400	400	Cost for service/maintenance.
6) Service and calibration of floor scale	2200	2200	Cost for calibration at approximately RM 1200/yr.
7) Electricity	39	0	Used for small compost packaging. Others are negligible.
8) Packaging of compost	4498	0	Estimated price at MYR 0.25 for each kg of compost
9) Compost analysis	1380	1380	Final compost analysis (MYR 60 per sample)
10) Labour cost	15000	15000	MYR 1500 for 10 months.
**Total operational costs**	**26921**	**22384**	

**(B) Fixed cost**			

1) Floor scale	5000	5000	Floor scale for weighing purpose (1.5 m (W) x 1.5 m (L)).
2) Thermocouple	1000	1000	Used for on-site compost temperature determination
3) Small scale	300	0	Used for small packaging process (for commercial sale).
4) Sealer	500	0	Used for small packaging process (for commercial sale).
5) Compost screening materials for storage/packaging	1000	1000	Used during compost finishing.
6) Jumbo Bag	500	500	Used for storage of compost. Jumbo bag size is 0.9 m (W) x 0.9 m (L) x 1.1 m (H).
7) Farm tools (scoop etc.)	2000	2000	Tools for manual handling on-site
**Total fixed cost**	**10300**	**9500**	

**(C) Revenue**			

1) Compost sales	44,975	26985	
**Total earnings**	**44,975**	**26985**	

**(D) Cost and revenue analysis**			

1) Operational Cost	26921	22384	
2) Fixed Cost	390	350	Normalised 10 years for the floor scale and 5 years for the others.
3) Contigencies	1366	1137	5% of total cost (production + fixed)
4) Total Cost	28677	23871	
5) Total Earnings	44,975	26,985	
6) Gross Return	16,298	3,114	
7) Net return	5,998	-6,386	
8) Benefit to cost ratio (BCR)	1.58	1.13	
9) Minimal cycles for return	1.66	3.5	

It could be seen from Table 5 that the total cost included the cost for the analysis of the operational cost and fixed cost. However, the fixed cost did not cover the cost of purchasing new equipment, such as skid steer loader and shredder nor account for an investment to develop new infrastructure, especially a shading area (open-air building) for composting. The scenario in MARDI illustrated that the equipment and infrastructure were readily available, therefore, any new investment was not required.

It was indicated from the analysis of the benefit-cost ratio (BCR) in Table 5 that composting was financially viable under both scenarios. To be specific, the BCR values, which were higher than 1, indicated that a scenario under a particular project offered more benefits compared to liabilities (Suhaimi et al., 2014). In this case, the project was deemed sustainable and could be expanded for implementation at a larger scale. However, certain elements should be present, such as readily available heavy machinery equipment and infrastructure, which would lower the implementation cost. Nevertheless, this aspect was not critical as many of the MARDI research stations nationwide were supplied with the equipment and infrastructure.

## 4 Conclusion

Composting of yard waste is feasible for continuous implementation of its practice in MARDI Headquarters, Serdang, Selangor, Malaysia. The data developed in this study offered the most crucial information on the amount of waste generated, which amounted to an average of 0.60 tonnes a week on the f.w. basis. These wastes were then formulated and mixed with livestock waste, functioning as an important nitrogen source to produce a stable compost at a C/N ratio of 10:1 to 15:1 after 60–90 days. Furthermore, it was suggested through other results of the compost, especially the germination test, that the results would be useful for the eventual application. Following that, this study proposed that yard waste was a high-carbon material, which was compostable through appropriate formulation and nitrogen addition. Meanwhile, the economic analysis found that a positive benefit-cost ratio (BCR) values higher than 1 suggested the viability of the project. Notably, the aerobic composting implemented in this study is practical for large composting. To illustrate, MARDI consists of eight main research stations and 24 support stations, covering a vast land area of 7,065 ha in Malaysia. Numerous annual and perennial crops, including landscape plant trees were developed in a large part of the research stations, contributing to an abundant source of biomass, which is practical for composting. Therefore, this approach is suitable for introduction as a potential approach at the institutional level, emphasising an efficient and effective method on yard waste management in MARDI research stations in the country.

## Declarations

### Author contribution statement

Mohammad Hariz Bin Abdul Rahman: Conceived and designed the experiments; Performed the experiments; Analyzed and interpreted the data; Contributed reagents, materials, analysis tools or data; Wrote the paper.

Tosiah Sadi: Conceived and designed the experiments.

Aimi Athirah Ahmad: Analyzed and interpreted the data; Contributed reagents, materials, analysis tools or data.

Intan Nadhirah Masri, Masnira Mohammad Yusoff, Hasliana Kamaruddin: Contributed reagents, materials, analysis tools or data.

Nur Alyani Shakri, Mohd Abhar Akmal Hamid, Rashidah Abdul Malek: Performed the experiments.

### Funding statement

This work was supported by the 10.13039/501100007702Malaysian Agricultural Research and Development Institute (MARDI) under projects no. FS017110 and 21003004030001-B.

### Competing interest statement

The authors declare no conflict of interest.

### Additional information

No additional information is available for this paper.

## References

[bib1] Adhikari B.K., Barrington S., Martinez J., King S. (2009). Effectiveness of three bulking agents for food waste composting. Waste Manag..

[bib2] Abdel-Shafy H.I., Mansour M.S. (2018). Solid waste issue: sources, composition, disposal, recycling, and valorization. Egypt. J. Petrol..

[bib3] A&L Canada Laboratories Inc (2004). Compost Management Program. http://www.alcanada.com/index_htm_files/Compost_Handbook.pdf.

[bib4] Awasthi M.K., Pandey A.K., Bundela P.S., Khan J. (2015). Co-composting of organic fraction of municipal solid waste mixed with different bulking waste: characterization of physicochemical parameters and microbial enzymatic dynamic. Bioresour. Technol..

[bib5] Baharuddin A.S., Wakisaka M., Shirai Y., Abd-Aziz S., Abdul Rahman N.A., Hassan M.A. (2009). Co-composting of empty fruit bunches and partially treated palm oil mill effluents in pilot scale. Int. J. Agric..

[bib6] Bernal M.P., Paredes C., Sanchew-Monedero M.A., Cegarra J. (1998). Maturity and stability parameters of composts prepared with a wide range of organic wastes. Bioresour. Technol..

[bib7] Boldrin A., Andersen J.K., Christensen T.H. (2011). Environmental assessment of garden waste management in the Municipality of Aarhus, Denmark. Waste Manag..

[bib8] Bong C.P.C., Goh R.K.Y., Lim J.S., Ho W.S., Lee C.T., Hashim H., Mansor N.N.A., Ho C.S., Ramli A.R., Takeshi F. (2017). Towards low carbon society in Iskandar Malaysia: implementation and feasibility of community organic waste composting. J. Environ. Manag..

[bib9] Bradstreet R.B. (1954). Kjeldahl method for organic nitrogen. Anal. Chem..

[bib10] Breitenbeck G.A., Schellinger D. (2004). Calculating the reduction in material mass and volume during composting. Compost Sci. Util..

[bib11] British Standards Institution (2011). PAS 100:2011 Specification for Composted Materials. http://www.wrap.org.uk/sites/files/wrap/PAS%20100_2011.pdf.

[bib12] Brown S. (2016). Greenhouse gas accounting for landfill diversion of food scraps and yard waste. Compost Sci. Util..

[bib13] Burnley S.J., Ellis J.C., Flowerdew R., Poll A.J., Prosser H. (2007). Assessing the composition of municipal solid waste in Wales. Resour. Conserv. Recycl..

[bib14] Cáceres R., Malińska K., Marfà O. (2018). Nitrification within composting: a review. Waste Manag..

[bib15] California Compost Quality Council (2001). Compost Maturity Index. http://compostingcouncil.org/wp/wp-content/uploads/2014/02/2-CCQC-Maturity-Index.pdf.

[bib16] Carlile W.R., Cattivello C., Zaccheo P. (2015). Organic growing media: constituents and properties. Vadose Zone J..

[bib17] Cesaro A., Belgiorno V., Guida M. (2015). Compost from organic solid waste: quality assessment and European regulations for its sustainable use. Resour. Conserv. Recycl..

[bib18] Chaves B., De Neve S., Boeckx P., Van Cleemput O., Hofman G. (2005). Screening organic biological wastes for their potential to manipulate the N release from N-rich vegetable crop residues in soil. Agric. Ecosyst. Environ..

[bib19] Cooperband L.R. (2002). The Art and Science of Composting. A Resource for Farmers and Compost Producers.

[bib20] Couth R., Trois C. (2012). Cost effective waste management through composting in Africa. Waste Manag..

[bib21] Davis J.G., Kendall P. (2005). Preventing E. coli from Garden to Plate. Food and Nutrition Series. Food Safety; No. 9.369. https://extension.colostate.edu/topic-areas/nutrition-food-safety%20health/preventing-e-coli-from-garden-to-plate-9-369/.

[bib22] Edjabou M.E., Jensen M.B., Götze R., Pivnenko K., Petersen C., Scheutz C., Astrup T.F. (2015). Municipal solid waste composition: sampling methodology, statistical analyses, and case study evaluation. Waste Manag..

[bib23] Fadzilah K., Saini H.S., Atong M. (2017). Physicochemical characteristics of oil palm frond (OPF) composting with fungal inoculants. Pertanika J. Trop..

[bib24] Fischer D., Glaser B., Kumar S., Bharti A. (2012). Synergisms between compost and biochar for sustainable soil amelioration. Management of Organic Waste.

[bib25] Fourti O., Jedidi N., Hassen A. (2013). Physico-chemical aspects during the composting of municipal solid wastes and sewage sludge in a semiindustrial composting plant. Afr. J. Microbiol. Res..

[bib26] Golueke C.G. (1991). Principles of composting. The Staff of BioCycle Journal of Waste Recycling. The Art and Science of Composting.

[bib27] Golueke C.G. (1991). Composting methods and operations. The Staff of BioCycle Journal of Waste Recycling. The Art and Science of Composting.

[bib28] Gopinathan M., Thirumurthy M. (2012). Evaluation of phytotoxicity for compost from organic fraction of municipal solid waste and paper & pulp mill sludge. Environ. Res. Eng. Manag..

[bib29] Grube M., Lin J.G., Lee P.H., Kokorevicha S. (2006). Evaluation of sewage sludge-based compost by FT-IR spectroscopy. Geoderma.

[bib30] Guo R., Li G., Jiang T., Schuchardt F., Chen T., Zhao Y., Shen Y. (2012). Effect of aeration rate, C/N ratio and moisture content on the stability and maturity of compost. Bioresour. Technol..

[bib31] Hachicha R., Rekik O., Hachicha S., Ferchichi M., Woodward S., Moncef N., Cegarra J., Mechichi T. (2012). Co-composting of spent coffee ground with olive mill wastewater sludge and poultry manure and effect of Trametes versicolor inoculation on the compost maturity. Chemosphere.

[bib32] Hartz T.K., Giannini C. (1998). Duration of composting of yard wastes affects both physical and chemical characteristics of compost and plant growth. HortScience.

[bib33] Haynes R.J., Belyaeva O.N., Zhou Y.F. (2015). Particle size fractionation as a method for characterizing the nutrient content of municipal green waste used for composting. Waste Manag..

[bib34] Hemalatha B. (2013). Comparative evaluation of biodegradability of yard waste and fruit waste with industrial effluents by vermicomposting. Int. J. Adv. Eng. Technol..

[bib35] Hermann B.G., Debeer L., De Wilde B., Blok K., Patel M.K. (2011). To compost or not to compost: carbon and energy footprints of biodegradable materials’ waste treatment. Polym. Degrad. Stabil..

[bib36] Hernández T., Chocano C., Moreno J.L., García C. (2016). Use of compost as an alternative to conventional inorganic fertilizers in intensive lettuce (Lactuca sativa L.) crops—effects on soil and plant. Soil Till. Res..

[bib37] Hirai M.F., Chanyasak V., Kubota H. (1983). A standard measurement for compost maturity. Biocycle.

[bib38] Hsu J.H., Lo S.L. (1999). Chemical and spectroscopic analysis of organic matter transformations during composting of pig manure. Environ. Pollut..

[bib39] Huang G.F., Wong J.W.C., Wu Q.T., Nagar B.B. (2004). Effect of C/N on composting of pig manure with sawdust. Waste Manag..

[bib40] Inbar Y., Hadar Y., Chen Y. (1993). Recycling of cattle manure: the composting process and characterization of maturity. J. Environ. Qual..

[bib41] Jakobsen S.T. (1995). Aerobic decomposition of organic wastes 2. Value of compost as a fertilizer. Resour. Conserv. Recycl..

[bib42] Kalantari S., Hatami S., Ardalan M.M., Alikhani H.A., Shorafa M. (2010). The effect of compost and vermicompost of yard leaf manure on growth of corn. Afr. J. Agric. Res..

[bib43] Kannan A., Upreti R.K. (2008). Influence of distillery effluent on germination and growth of mung bean (Vigna radiata) seeds. J. Hazard Mater..

[bib44] Kuba T., Tschöll A., Partl C., Meyer K., Insam H. (2008). Wood ash admixture to organic wastes improves compost and its performance. Agric. Ecosyst. Environ..

[bib45] Kulikowska D., Sindrewicz S. (2018). Effect of barley straw and coniferous bark on humification process during sewage sludge composting. Waste Manag..

[bib46] Leu A. (2007). Organics and soil carbon: increasing soil carbon, crop productivity and farm profitability. Managing the Carbon Cycle’Katanning Workshop.

[bib47] Li Y., Park S.Y., Zhu J. (2011). Solid-state anaerobic digestion for methane production from organic waste. Renew. Sustain. Energy Rev..

[bib48] Li Q., Wang X.C., Zhang H.H., Shi H.L., Hu T., Ngo H.H. (2013). Characteristics of nitrogen transformation and microbial community in an aerobic composting reactor under two typical temperatures. Bioresour. Technol..

[bib49] Lin C. (2008). A negative-pressure aeration system for composting food wastes. Bioresour. Technol..

[bib50] Liu C., Hofstra N., Franz E. (2013). Impacts of climate change on the microbial safety of pre-harvest leafy green vegetables as indicated by Escherichia coli O157 and Salmonella spp. Int. J. Food Microbiol..

[bib51] Luo Y., Liang J., Zeng G., Chen M., Mo D., Li G., Zhang D. (2018). Seed germination test for toxicity evaluation of compost: its roles, problems and prospects. Waste Manag..

[bib52] Maeda K., Hanajima D., Toyoda S., Yoshida N., Morioka R., Osada T. (2011). Microbiology of nitrogen cycle in animal manure compost. Microb. Biotechnol..

[bib53] Mary O., Hayford O., Matilda D., Richard T., Gregory K., Nanam D., Deborah M., Juanita P., Anton S. (2014). Heavy metal and proximate composition associated with the composting of cassava (Manihot esculenta) peels used in the cultivation of mushrooms in Ghana. Afr. J. Biotechnol..

[bib54] Mathur S.P., Owen G., Dinel H., Schnitzer M. (1993). Determination of compost biomaturity. I. Literature review. Biol. Agric. Hortic..

[bib55] Mkhabela M.S., Warman P.R. (2005). The influence of municipal solid waste compost on yield, soil phosphorus availability and uptake by two vegetable crops grown in a Pugwash sandy loam soil in Nova Scotia. Agric. Ecosyst. Environ..

[bib56] Michel F.C., Forney L.J., Huang A.J.F., Drew S., Czuprenski M., Lindeberg J.D., Reddy C.A. (1996). Effects of turning frequency, leaves to grass mix ratio and windrow vs. pile configuration on the composting of yard trimmings. Compost Sci. Util..

[bib57] Mohammad Hariz A.R., Ong H.K., Nurul Ain A.B., Fauzi J. (2013). Application of agro-waste compositional data to predict composting efficiency. J. Trop. Agric. Food Sci..

[bib58] Moqsud M.A., Bushra Q.S., Rahman M.H. (2011). Composting barrel for sustainable organic waste management in Bangladesh. Waste Manag. Res..

[bib59] Onifade A.K., Inegbu U.O., Fadipe D.O. (2015). Comparing the antibiotics susceptibility pattern of Escherichia coli isolated from feacal samples of human, cattle and chicken in Ondo state, Nigeria. Annals Pharm. Res..

[bib60] Pace M.G., Miller B.E., Farrel-Po K.L. (1995). The Composting Process. https://digitalcommons.usu.edu/cgi/viewcontent.cgi?article=1047&context=extension_histall.

[bib61] Pan I., Dam B., Sen S.K. (2012). Composting of common organic wastes using microbial inoculants. 3. Biotech.

[bib62] Pandey P.K., Cao W., Wang Y., Vaddella V., Castillo A.R., Souza A., del Rio N.S. (2016). Simulating the effects of mesophilic anaerobic and aerobic digestions, lagoon system, and composting on pathogen inactivation. Ecol. Eng..

[bib63] Promila K., Kumar S. (2000). Vigna radiata seed germination under salinity. Biol. Plantarum.

[bib64] Rasapoor M., Nasrabadi T., Kamali M., Hoveidi H. (2009). The effects of aeration rate on generated compost quality, using aerated static pile method. Waste Manag..

[bib65] Richard T., Trautmann N. (1996). C/N Ratio.

[bib66] Rosenani A.B., Rovica R., Cheah P.M., Lim C.T. (2016). Growth performance and nutrient uptake of oil palm seedling in prenursery stage as influenced by oil palm waste compost in growing media. Int. J. Agron..

[bib67] Rupani P.F., Embrandiri A., Ibrahim M.H., Shahadat M., Hansen S.B., Mansor N.N.A. (2017). Bioremediation of palm industry wastes using vermicomposting technology: its environmental application as green fertilizer. 3 Biotech.

[bib68] Sabki M.H., Lee C.T., Bong C.P., Klemes J.J. (2018). A review on the economic feasibility of composting for organic waste management in Asian countries. Chem. Eng. Trans..

[bib69] Salehi M., Ebrahimi R., Maleki A., Mobtaker H.G. (2014). An assessment of energy modeling and input costs for greenhouse button mushroom production in Iran. J. Clean. Prod..

[bib70] Samsudina M.D.M., Dona M.M. (2013). Municipal solid waste management in Malaysia: current practices, challenges and prospect. Hospital.

[bib71] Shahudin Z., Zain S.M., Basri N.E.A., Zaini N.S.M., Saad N.F.M., Basri H. (2013). Preliminary study for designing a yard waste composting facility in Universiti Kebangsaan Malaysia. J. Teknol..

[bib72] Shi Y., Ge Y., Chang J., Shao H., Tang Y. (2013). Garden waste biomass for renewable and sustainable energy production in China: potential, challenges and development. Renew. Sustain. Energy Rev..

[bib73] Solaiman Z.M., Yang H.J., Archdeacon D., Tippett O., Tibi M., Whiteley A.S. (2019). Humus-rich compost increases lettuce growth, nutrient uptake, mycorrhizal colonisation, and soil fertility. Pedosphere.

[bib74] Soobhany N., Mohee R., Garg V.K. (2017). Inactivation of bacterial pathogenic load in compost against vermicompost of organic solid waste aiming to achieve sanitation goals: a review. Waste Manag..

[bib75] Suhaimi M.Y., Mohamad A.M., Hani M.N.F. (2014). Potential and viability analysis for ginger cultivation using fertigation technology in Malaysia. Int. J. Innovat. Appl. Stud..

[bib76] Tarmudi Z., Abdullah M.L., Tap A.O.M. (2012). A review of municipal solid waste management in Malaysia. J. Teknol..

[bib77] Thyagarajan D., Barathi M., Sakthivadivu R. (2013). Scope of poultry waste utilization. IOSR J. Agric. Vet. Sci..

[bib78] Tibu C., Annang T.Y., Solomon N., Yirenya-Tawiah D. (2019). Effect of the composting process on physicochemical properties and concentration of heavy metals in market waste with additive materials in the Ga West Municipality, Ghana. Int. J. Recycl. Org. Waste Agric..

[bib79] Tiquia S.M., Tam N.F.Y., Hodgkiss I.J. (1996). Microbial activities during composting of spent pig-manure sawdust litter at different moisture contents. Bioresour. Technol..

[bib80] Tiquia S.M., Richard T.L., Honeyman M.S. (2000). Effect of windrow turning and seasonal temperatures on composting of hog manure from hoop structures. Environ. Technol..

[bib81] Tiquia S.M., Tam N.F.Y. (2000). Co-composting of spent pig litter and sludge with forced-aeration. Bioresour. Technol..

[bib82] Trautmann N., Richard T., Krasny M. (1996). The Science and Engineering of Composting. http://compost.css.cornell.edu/monitor/monitortemp.htmi.

[bib83] Van Fan Y., Lee C.T., Klemeš J.J., Bong C.P.C., Ho W.S. (2016). Economic assessment system towards sustainable composting quality in the developing countries. Clean Technol. Environ..

[bib84] Vasileva V., Kostov O. (2015). Effect of mineral and organic fertilization on alfalfa forage and soil fertility. Emir. J. Food Agric..

[bib85] Vigneswaran S., Kandasamy J., Johir M.A.H. (2016). Sustainable operation of composting in solid waste management. Procedia Environ. Sci..

[bib86] Walker D.J., Clemente R., Bernal M.P. (2004). Contrasting effects of manure and compost on soil pH, heavy metal availability and growth of Chenopodium album L. in a soil contaminated by pyritic mine waste. Chemosphere.

[bib87] Walkley A., Black I.A. (1934). An examination of the Degtjareff method for determining soil organic matter, and a proposed modification of the chromic acid titration method. Soil Sci..

[bib88] Wang K., He C., You S., Liu W., Wang W., Zhang R., Qi H., Ren N. (2015). Transformation of organic matters in animal wastes during composting. J. Hazard Mater..

[bib89] Waqas M., Nizami A.S., Aburiazaiza A.S., Barakat M.A., Ismail I.M.I., Rashid M.I. (2017). Optimization of food waste compost with the use of biochar. J. Environ. Manag..

[bib90] Wei L., Shutao W., Jin Z., Tong X. (2014). Biochar influences the microbial community structure during tomato stalk composting with chicken manure. Bioresour. Technol..

[bib91] Whitehead D., Tinsley J. (1963). The biochemistry of humus formation. J. Sci. Food Agric..

[bib92] Wichuk K.M., McCartney D. (2013). Compost stability and maturity evaluation - a literature review. J. Environ. Eng. Sci..

[bib93] Wong J.W.C., Mak K.F., Chan N.W., Lam A., Fang M., Zhou L.X., Wu Q.T., Liao X.D. (2001). Co-composting of soybean residues and leaves in Hong Kong. Bioresour. Technol..

[bib94] Yazdani R., Barlaz M.A., Augenstein D., Kayhanian M., Tchobanoglous G. (2012). Performance evaluation of an anaerobic/aerobic landfill-based digester using yard waste for energy and compost production. Waste Manag..

[bib95] Yoshida H., Gable J.J., Park J.K. (2012). Evaluation of organic waste diversion alternatives for greenhouse gas reduction. Resour. Conserv. Recycl..

[bib96] Zang B., Li S., Michel F., Li G., Luo Y., Zhang D., Li Y. (2016). Effects of mix ratio, moisture content and aeration rate on sulfur odor emissions during pig manure composting. Waste Manag..

[bib97] Zhang D., Luo W., Li Y., Wang G., Li G. (2018). Performance of co-composting sewage sludge and organic fraction of municipal solid waste at different proportions. Bioresour. Technol..

[bib98] Zulkepli N.E., Muis Z.A., Mahmood N.A.N., Hashim H., Ho W.S. (2017). Cost benefit analysis of composting and anaerobic digestion in a community: a review. Chem. Eng. Trans..

