# Corrigendum to “Inventory and composting of yard waste in Serdang, Selangor, Malaysia” [Heliyon 6 (7) (July 2020) 04486]

**DOI:** 10.1016/j.heliyon.2020.e04748

**Published:** 2020-08-25

**Authors:** Mohammad Hariz Abdul Rahman, Tosiah Sadi, Aimi Athirah Ahmad, Intan Nadhirah Masri, Masnira Mohammad Yusoff, Hasliana Kamaruddin, Nur Alyani Shakri, Mohamad Abhar Akmal Hamid, Rashidah Ab. Malek

**Affiliations:** a Agrobiodiversity & Environment Research Centre, MARDI, 43400 Serdang, Selangor, Malaysia; b Soil & Fertilizer Research Centre, MARDI, 43400 Serdang, Selangor, Malaysia; c Socio Economic, Market Intelligence & Agribusiness Research Center, MARDI, 43400 Serdang, Selangor, Malaysia; d Horticulture Research Centre, MARDI, 43400 Serdang, Selangor, Malaysia

In the original published version of this article, the authors incorrectly referred to dry weight instead of fresh weight in three locations: the abstract, the results and discussion (section “Inventory of waste and design of compost processing facility”), and the conclusion. The authors apologise for the oversight. The following three sentences have now been corrected:

“The total amount of yard waste generated from June 2017 to December 2017 was 16.75 tonnes with an average generation of 0.60 tonnes per week on the fresh weight (f.w.) basis.”

“The total yard waste generated from June to December 2017 amounted to 16.75 tonnes with an average of 0.60 tonnes evaluated per week on the fresh weight (f.w.) basis.”

“The data developed in this study offered the most crucial information on the amount of waste generated, which amounted to an average of 0.60 tonnes a week on the f.w. basis.”

Both the HTML and PDF versions of the article have been updated to correct the error.

